# Influence of Interfacial Reactions on Perovskite Optoelectronic Devices

**DOI:** 10.1002/smtd.202500438

**Published:** 2025-05-03

**Authors:** Zhongcheng Yuan, Sai Bai, Feng Gao, Henry J. Snaith

**Affiliations:** ^1^ Clarendon Laboratory Department of Physics University of Oxford Oxford OX1 3PU UK; ^2^ Institute of Fundamental and Frontier Sciences University of Electronic Science and Technology of China Chengdu 611731 China; ^3^ Department of Physics, Chemistry and Biology Linköping University Linköping 58330 Sweden

**Keywords:** deprotonation, film growing control, interfacial reactions, light‐emitting diodes, metal halide perovskite

## Abstract

Interfacial materials tend to alter the crystallization, films growth and defect formation process of the as‐deposited perovskites, which has been a critical and fundamental factor in determining the efficiency and operational stability of perovskite‐based optoelectronic devices. This review explores the underlying mechanism of interfacial reactions, which can either result in degradations or be beneficial. The influence of interfacial reactions, mainly interface‐induced deprotonation of organic cations and amidation processes, are discussed in relation to their impact on perovskite film growth and ensuing optoelectronic device performance. It is further proposed strategies to regulate these reactions and mitigate their negative effects to achieve high performance optoelectronic devices.

## Graphical abstract

Interface‐induced chemical reactions are commonly observed in perovskite‐based optoelectronic devices. This review summarizes the main types of these reactions‐primarily deprotonation and amidation, and highlights their beneficial roles in regulating perovskite film crystallization, growing processes, defect formation, and overall device performance. Rather than being avoided, these reactions should be recognized as valuable tools for advancing the development of highly stable perovskite optoelectronic devices.

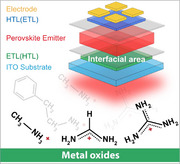

## 1 Introduction

Metal halide perovskites are promising materials for high‐performance and low‐cost optoelectronic devices for various applications. In the last decade, innovations in perovskite film growth control and fine defect passivation have greatly promoted the development of high‐performance optoelectronic devices, including photovoltaic solar cells (PSCs)^[^ ^1^, ^2^, ^3^ ^]^ light‐emitting diodes (LEDs),^[^ ^4^, ^5^, ^6^, ^7^, ^8^ ^]^ photodetectors^[^ ^9^, ^10^ ^]^ and X‐ray detectors, etc.^[^ ^11^, ^12^, ^13^ ^]^ However, the perovskites, especially organic‐inorganic hybrid perovskites, are chemically unstable when exposing to ultraviolet (UV) light, oxygen, moisture, or under electrical field, which has been a critical concern for their widespread deployment.^[^ ^14^, ^15^, ^16^, ^17^, ^18^, ^19^, ^20^, ^21^, ^22^, ^23^ ^]^ Despite these factors, direct contacts of perovskites with charge transporting materials (CTMs) further complicates the perovskite chemical stability issues.^[^ ^17^, ^24^ ^]^ Interfaces between the metal halide perovskites and CTMs play important roles in determining contact quality. The hydrophilicity, morphology, and temperature of the substrates all have direct impacts on the perovskite crystallization, leading to different morphology, crystallinity and defect densities, *etc*. of the perovskite films.^[^ ^25^, ^26^, ^27^, ^28^, ^29^, ^30^ ^]^ This further alters charge carriers transport and recombination in the films, and ensuing device performance.^[^ ^31^, ^32^, ^33^ ^]^ Additionally, chemical reactions led to relatively weak stability of perovskites on reactive metal oxides.^[^ ^34^ ^]^ It is revealed that under continuous UV light illumination, free‐radicals (superoxide (O_2_^‐^)) generated in the metal oxide layer, accelerate the decomposition of perovskites through reacting with the organic cations.^[^ ^16^, ^17^, ^20^ ^]^ This is a critical pathway for perovskite degradation considering PSCs are mainly used outdoor and UV light cannot be ignored.^[^ ^20^ ^]^

While perovskite LEDs (PeLEDs) are primarily deployed indoors, and the‐state‐of‐the‐art encapsulation techniques will greatly minimize the influence from UV light, moisture and oxygen. Considering most metal halide perovskites contain organic cations, like formamidinium (FA^+^), methylammonium (MA^+^), guanidium (GUA^+^), or long chain organic cations including phenethylammonium (PEA^+^), butylammonium (BA^+^), etc., in low dimensional perovskites, which all show certain chemical reactivity.^[^ ^35^, ^36^, ^37^ ^]^ When directly depositing these organic‐inorganic hybrid perovskites on commonly used substrates like zinc oxide (ZnO), tin oxide (SnO_2_), nickel oxide (NiO_x_) and molybdenum trioxide (MoO_3_), interface‐induced chemical reactions can lead to decomposition of the perovskites.^[^ ^38^, ^39^, ^40^, ^41^, ^42^, ^43^, ^44^, ^45^, ^46^, ^47^, ^48^ ^]^ However, different from the view that interfacial reactions are detrimental to device performance, especially the operational stability of PSCs,^[^ ^34^, ^47^, ^48^, ^49^, ^50^ ^]^ it turns out that the interfacial reactions play a remarkably positive role for achieving highly emissive perovskite films for PeLEDs.^[^ ^31^, ^36^, ^37^ ^]^ Therefore, focusing on the interfacial reactions and revealing the mechanism can be extremely valuable for obtaining high‐performance perovskite optoelectronic devices, including PSCs and PeLEDs.^[^ ^38^, ^51^ ^]^

In this review, we first summarize the mechanism of interface‐included reactions, especially from underlying interfaces, where perovskite films are deposited. We focus on interface‐induced deprotonation of the organic cations, which has a significant impact upon the compositions and qualities of the perovskite films. We further reveal the influence of this deprotonation upon device performance of PeLEDs and outline strategies for controlling the interfacial reaction rates with A‐site cation engineering and interface modifications. Furthermore, we include a consideration of other interfacial reactions like amidation and reactions happening between processing additives in perovskites and interfacial modification layers. Finally, we give a perspective on leveraging interfacial reactions to achieve stable perovskite films for other optoelectronic devices. We believe interfacial reactions could be exceptionally useful factors to promote the development of highly stable perovskite‐based optoelectronics devices and should not be considered something to “avoid at all cost”, as is common philosophy.

## 2 Basics of Perovskites and PeLEDs

**Figure 1:**
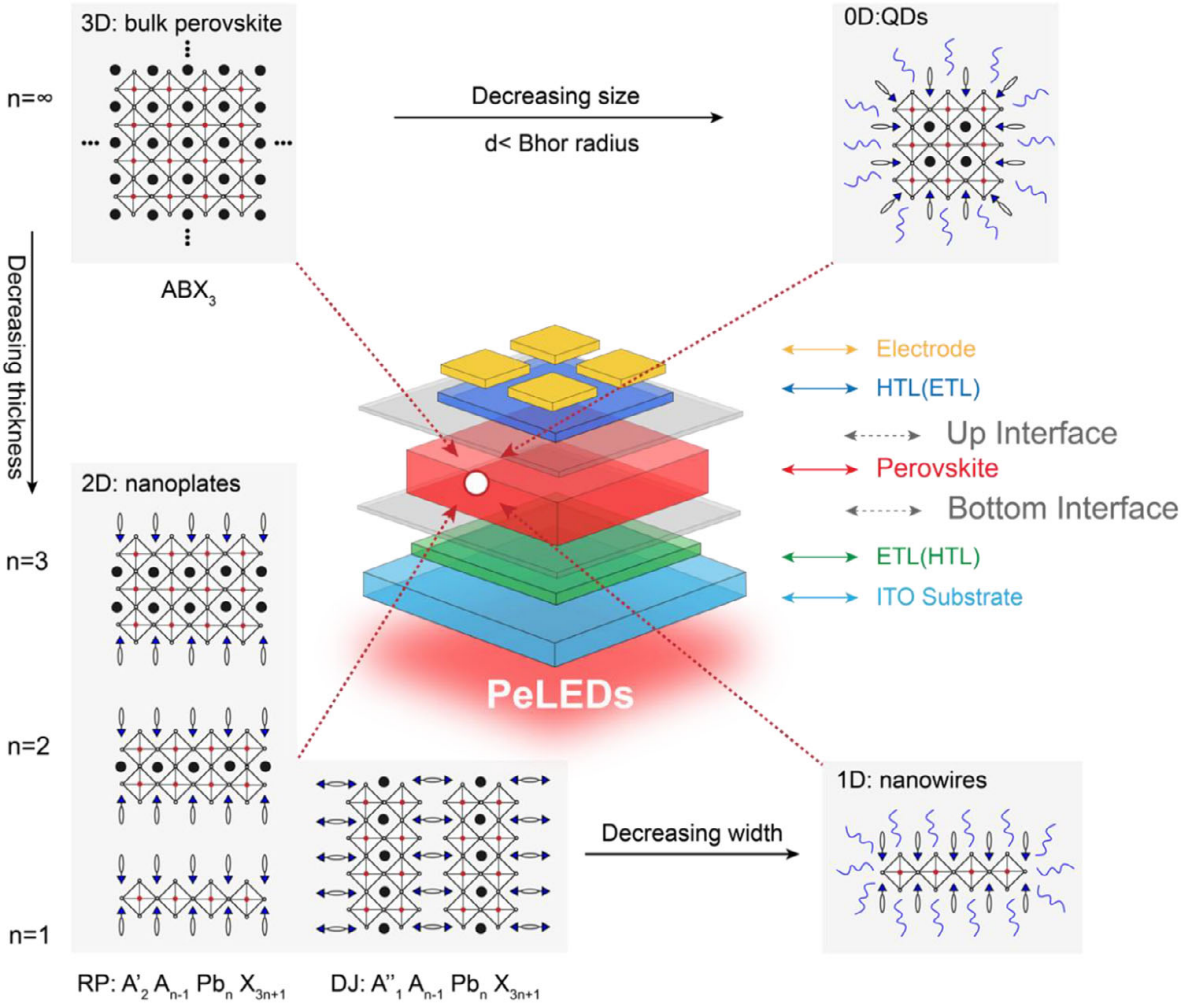
Illustration of 3D, 2D (nanoplates), 1D (nanowires) and 0D (quantum dots) perovskites crystal structure that used as emitters in perovskite optoelectronic devices. Inset is a schematic illustration of a typical PeLED device structure. The interfaces between perovskite and charge transporting layers are labelled in grey.

Perovskite represents a basic crystal structure with a chemical formula of ABX_3_, in which A is monovalent cation (for example, MA^+^, FA^+^ or Cs^+^ and B is divalent metal cation (Pb^2+^ or Sn^2+^) and X is halogen anions (I^‐^, Br^‐^ or Cl^‐^).^[^ ^52^ ^]^ A typical 3D perovskite structure is formed from sharing halides at [BX_6_]^4‐^ octahedral corner, where Pb^2+^ cations sitting in the octahedral centre and monovalent cations staying at the voids between these octahedrals (**Figure** **1**).^[^ ^53^ ^]^

Introducing long chain spacer, like aliphatic or aromatic alkylammonium cations, into perovskites contributes to the growth of 2D perovskites with a formula of A’_2_A_(n‐1)_M_n_X_(3n+1)_, where A’ is spacer cations that isolate perovskite layer and n is the number of lead halides octahedral layers between the spacers.^[^ ^54^, ^55^ ^]^ This brings more possibilities for tuning perovskite structures and optical properties (Figure 1).^[^ ^52^, ^56^, ^57^, ^58^ ^]^ However, solution processed 2D perovskite films commonly form layered perovskites with varying n values, also known as quasi‐2D (Q‐2D) perovskites or perovskites with multiple‐quantum wells.^[^ ^54^, ^55^, ^59^ ^]^ Another way to obtain low‐dimensional perovskites is synthesizing 2D perovskite nanoplates,^[^ ^60^, ^61^, ^62^ ^]^ 1D perovskite nanorods or nanowire^[^ ^63^, ^64^, ^65^ ^]^ and 0D perovskite quantum dots (QDs) via wet chemistry synthesis.^[^ ^66^, ^67^, ^68^ ^]^ By tuning the chemical compositions and dimensionality of the perovskite emitters, their emission wavelength can be continuously tuned from ultraviolet to near infrared (NIR) region (≈400–1000 nm).^[^ ^67^, ^69^, ^70^, ^71^ ^]^

A typical PeLED device with a sandwich structure is inserted in Figure 1, which has been well developed in other light‐emitting devices, like organic LEDs (OLEDs) and QDs LEDs (QLEDs).^[^ ^70^, ^72^ ^]^ The devices are commonly prepared on glass substrates with pre‐patterned transparent indium tin oxide (ITO). A perovskite emissive film is deposited between an electron transporting layer (ETL) and a hole transporting layer (HTL) by spin‐coating, blade coating or vacuum deposition etc. While high‐conductive metals, like silver, gold and aluminium are used as top opaque electrodes by high‐vacuum thermal evaporation.

## 3 Effects of Interfacial Reactions on Perovskites Growth

Among all the interfaces in perovskite optoelectronic devices, the interfaces between perovskites and CTMs are the most reactive, which critically affect the quality of perovskite films and device performance. The locations of interfacial reactions discussed in this review are labelled in grey in the device structure (Figure 1).

### 3.1 Interface Induced Deprotonation of Organic Cations

**Figure 2:**
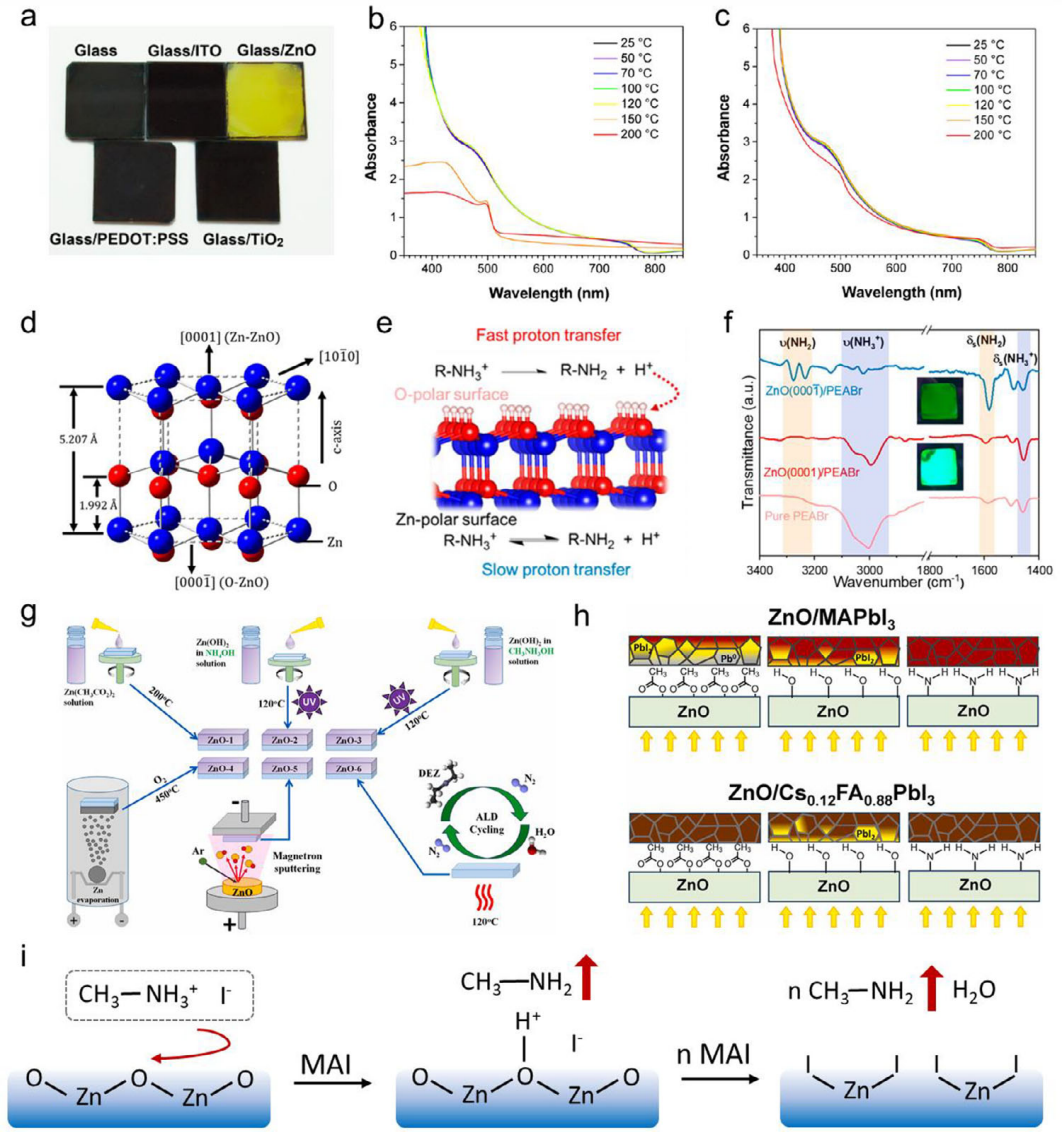
a) Photographs of MAPbI_3_ films deposited on different substrates after thermal annealing process; b,c) Ultraviolet‐visible (UV–vis) spectroscopy of MAPbI_3_ films deposited on ZnO and ITO substrates that annealed for different duration. Reproduced with permission.^[^ ^34^ ^]^ Copyright 2015, American Chemical Society; d) crystal structure of wurtzite ZnO; e) Schematic illustration of the deprotonation of PEA^+^ on ZnO surface with Zn‐ and O‐polar facets; f) Fourier transform infrared spectroscopy (FTIR) spectra of PEABr films deposited on ZnO with Zn‐ and O‐polar facets. Reproduced with permission.^[^ ^79^ ^]^ Copyright 2022, American Chemical Society; g) ZnO films prepared with different routes, including sol‐gel process, vacuum depositions and ALD; h) Chemcial stablity of MAPbI_3_ and Cs_0.12_FA_0.88_PbI_3_ films deposited on acetate‐, hydroxyl‐, and amine‐terminated ZnO surfaces. Reproduced with permission.^[^ ^80^ ^]^ Copyright 2025, Elsevier Ltd.; i) Schematic mechanism of ZnO induced deprotonation process of MA^+^.

ZnO possesses impressive carrier mobility, high conductivity and suitable energy level and has been verified as an ideal electron transporting material for high‐performance optoelectronic devices.^[^ ^72^, ^73^, ^74^, ^75^, ^76^, ^77^ ^]^ Kelly and co‐workers observed that MAPbI_3_ films showed distinct chemical stability on different substrates (**Figure** **2**a–c).^[^ ^34^, ^42^ ^]^ ZnO substrate exhibited the highest reactivity when contacting with MAPbI_3_ films and changed the perovskite film color from dark brown to yellow during thermal annealing process.^[^ ^78^ ^]^ Further results suggested that basic feature of ZnO surface triggered the proton‐transfer reactions of MA^+^ at the interface, leading to the formation of volatile MA molecules and crystalized PbI_2_ in the films (Figure 2a).^[^ ^34^, ^39^ ^]^ The ZnO‐induced deprotonation process was further verified in another work by mixing ZnO with MAPbI_3_ powder. The gas generated during heating process was purged into a 2, 4 dinitrotoluene solution, which is a methylamine indictor. It was observed that the 2, 4 dinitrotoluene solution changed to blue, suggesting the formation of MA gas during the chemical reaction.^[^ ^49^ ^]^

Furthermore, it was revealed that the crystal facet of ZnO crystal played an important role in influencing the deprotonation rate of organic cations. ZnO had a wurtzite crystal structure, with O and Zn atoms stacking along the c‐axis, as shown in Figure 2d. By etching single crystal ZnO, Bai et.al. obtained (000$\bar{1}$) polar surface terminated with Zn and O atoms, respectively. It discovered that the (000$\bar{1}$) O polar surface showed a higher deprotonation rate of PEA^+^, resulting in obviously faster decomposition of PEABr in the Quasi‐2D PEA_2_Cs_n−1_Pb_n_Br_3n+1_ films and much decreased film emission intensity (Figure 2e,f).^[^ ^79^ ^]^

Additionally, surface ligands on ZnO surface showed different chemical reactivity when contacting with MAPbI_3_ films. Troshin and co‐workers prepared ZnO film with six different routes to tune the surface chemical states: three of them were deposited by solution processes and the rest by thermal evaporation, magnetron sputtering and atomic layer deposition (ALD), respectively (Figure 2g).^[^ ^80^ ^]^ MAPbI_3_ films displayed the fastest degradation on ZnO films terminating with acetate ligands (Ac^‐^) and hydroxyl groups (OH^‐^), suggesting these ligands accelerated the deprotonation process.^[^ ^81^ ^]^ However, it was interesting to note that the degradation of MAPbI_3_ films was quite hindered by ZnO film with terminal ‐NH_2_ or ‐NHCH_3_ groups. The much enhanced chemical stability compared to the ones on bare glasses suggested that these ZnO layers could be more suitable ETLs for highly stable PSC with delicate optimization.^[^ ^80^ ^]^ Furthermore, ZnO nanoparticle (ZnO NP) films annealed at high temperature, which removed part of the surface ligands, or adding modification layers on top of the ZnO films weakened the ability of ZnO to deprotonate MA^+^, leading to improved films stability.^[^ ^38^, ^39^ ^]^ These results suggested surface ligands of ZnO NP or thin films, like surface OH^‐^ and residual Ac^‐^, tend to promote/accelerate the deprotonation process of the A‐site cations (Figure 2h).^[^ ^34^, ^39^ ^]^

The mechanism of ZnO‐induced deprotonation of MA^+^ is summarized in Figure 2i. The basic feature of ZnO deprotonates MA^+^ at the interface and generates MA gas during the heating process. This interfacial reaction transfers ZnO to ZnI_2_ at the interface, which has been verified by X‐ray diffraction (XRD), and X‐ray photoelectron spectroscopy (XPS) in several publications.^[^ ^40^, ^49^, ^82^, ^83^ ^]^ The basic surface of ZnO films is the origin of the deprotonation process of A‐site organic cations, and the ability to deprotonate organic cations could be well controlled by modifying ZnO films with different surface ligands or introducing certain surface treatments.

Similar deprotonation process was also reported for perovskite films deposited on other metal oxides, like NiO_x_ and SnO_2_ substrates.^[^ ^40^, ^84^ ^]^ For example, McGehee and co‐workers noted that the Ni^3+^ in the NiO_x_ films served as a proton acceptor and was capable to deprotonate the organic cations (MA^+^, FA^+^ etc.). In addition to the deprotonation reaction, the NiO_x_ films further oxidized the iodine species in the perovskites and resulted in perovskite film decomposition.^[^ ^40^ ^]^ To stabilize the perovskite films, thin modification layers, for example, nickel acetate, self‐assembled monolayers (SAMs)‐[4‐(3,6‐dimethyl‐9*H*‐carbazol‐9‐yl)butyl]phosphonic acid (Me‐4PACz) were coated on NiO_x_ layer to inhibit the interfacial reactions.^[^ ^85^, ^86^ ^]^

### 3.2 Parameters Influencing Interface‐Induced Deprotonation Process

Since the interface‐induced deprotonation process is basically an acid‐based reaction, which is critically associated with the acidity of A‐site cations and the alkaline substrates. Acid dissociation constant (pKa) values of A‐site cations and isoelectric point (IEP) of CTMs are the quantitative values used to assess the reactivity between them.

#### 3.2.1 pKa Values of Organic Cations

To systematically study the deprotonation process of A‐site cations, we use pKa, a value to describe the strength of an acid in a solution, to describe their ability to donate a proton. For a typical acid dissociation reaction,

(1) $$A^{+}\to A^{0}+H^{+}$$

In which [A^0^], [H^+^], [A^+^] represent the concentrations of deprotonated product (A^0^), H^+^ and cations (A^+^), respectively. The pKa can be defined by

(2) $$pKa\left(A^{+}\right)=-\log\frac{\left[A^{0}\right]\left[H^{+}\right]}{\left[A^{+}\right]}$$

A lower pKa represents a higher acidity and a higher ability to be deprotonated.^[^ ^35^ ^]^ **Table** **1** lists pKa values of commonly used A‐site organic cations.

**Table 1:** pKa values of A‐site cations and the IEP of commonly used metal oxides in optoelectronic devices.

CTLs	IEP	A‐site cations	pKa
SnO_2_	7.3	FA^+^	11.5
TiO_2_	4.7‐6.2	MA^+^	10.66
ZrO_2_	6.7	PEA^+^	9.83
Al_2_O_3_	5.6‐9.2	BA^+^	10.6
NiO	10.3 ±0.4	DMA^+^	10.73
ZnO	9.2‐10.3	GUA^+^	13.6
MgO	12.4 ±0.3	EA^+^	10.65

If no further reference, the IEP and pKa data in the table are referenced from here.^[^ ^35^, ^87^ ^]^

#### 3.2.2 Isoelectric Point of Metal Oxides

The acidic or basic surface nature of metal oxides could be characterized by their IEP, which is the PH of that a solid submerged in a liquid carries no net electrical charge. We list the IEP of commonly used metal oxides in Table 1 according to previous publications.

Choosing suitable perovskite compositions and CTMs are of critical importance for controlling these interfacial reactions. It is suggested utilizing A‐site cations with higher pKa values and CTMs with a lower IEP would decrease the chemical reactivity between them.^[^ ^35^ ^]^

### 3.3 Synergistic Effect of Stoichiometry and Interfacial Reactions

Interfacial reactions are believed to be tightly associated with the operational stability of PSCs. In high‐performance PSCs, perovskite films are commonly prepared stoichiometrically or less stoichiometrically (lead halides are 5%–10% more than the organic halides) in the precursors, so that perovskite films showing high conductivity, high crystallinity and large grains could form.^[^ ^88^, ^89^, ^90^ ^]^ The interface‐induced loss of A‐site cations tends to create more lattice defects of perovskite crystals at the interface area during film growing or device operation, bringing in non‐radiative recombination centers and channels for ion migration.^[^ ^90^, ^91^, ^92^ ^]^ Furthermore, these interfacial reactions accelerate at a higher temperature or under light illumination, which speeds up the loss of A‐site cations and deteriorates device operational stability.^[^ ^16^, ^37^, ^93^, ^94^ ^]^

Very different from PSCs, using excessive A‐site cations, which we called “over‐stoichiometric” strategies are now widely used in high‐performance PeLEDs.^[^ ^5^, ^6^, ^7^, ^55^, ^95^ ^]^ It is believed that lattice defects, like vacancies of halides, interstitials of A‐site cations and antisite halides, show relatively low formation energy and contribute to serious non‐radiative recombination.^[^ ^96^ ^]^ Introducing extra A‐site cation halides could greatly improve the point defects formation energy, leading to a much decreased defect density in the perovskite films.^[^ ^37^, ^97^, ^98^ ^]^ Additionally, the over‐stoichiometric strategy modified the precursor states of the salts, and at higher stoichiometric conditions, smaller colloidal sizes formed in the precursor solution, which could be the main reason for the uniform grain size distribution in perovskite films.^[^ ^37^, ^97^ ^]^

**Figure 3:**
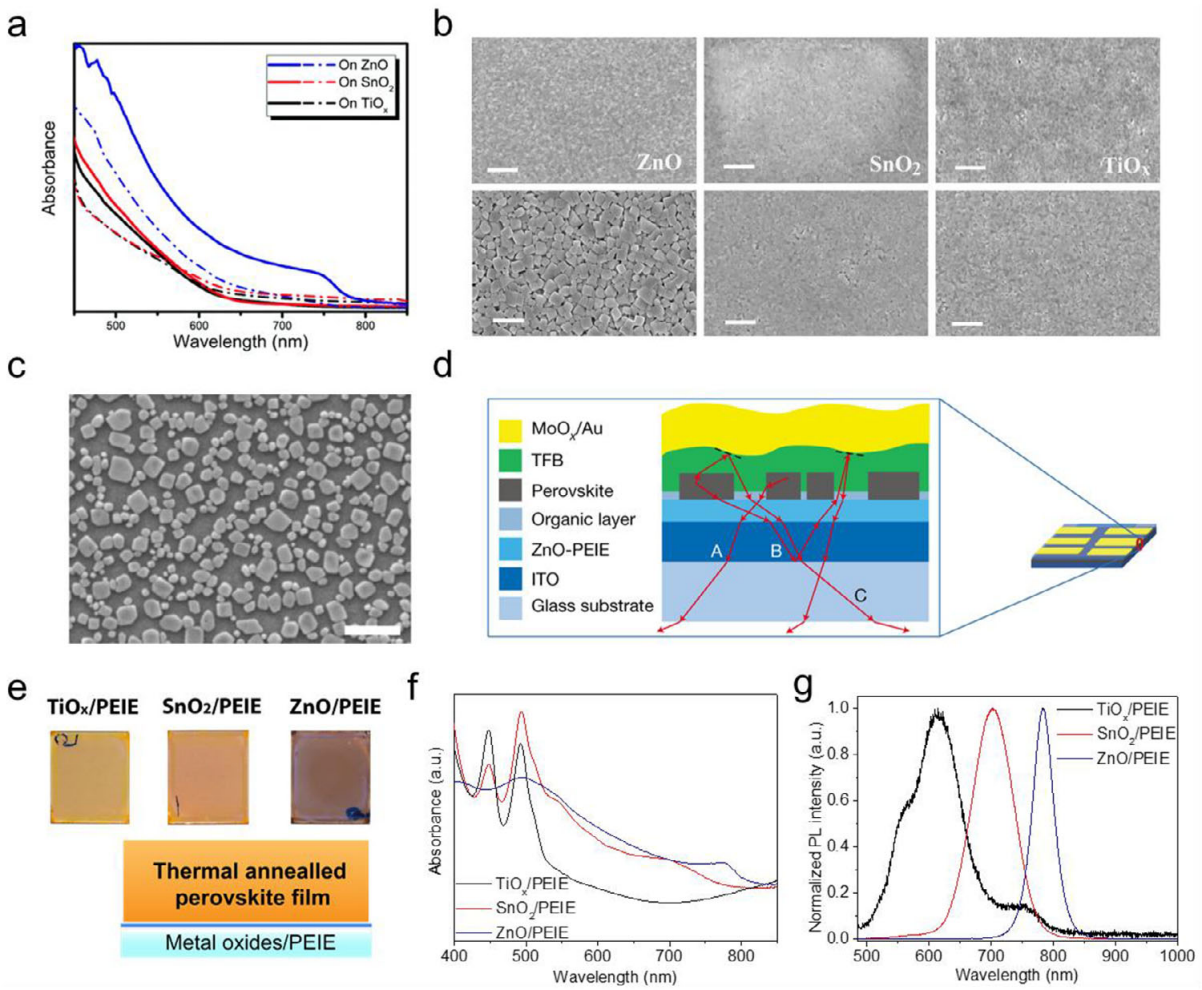
a) UV–vis absorption spectra of MAPbI_3_ films deposited on different metal oxide before (dash line) and after (solid line) vacuum drying process; b) SEM images of MAPbI_3_ films deposited on different metal oxide substrates before (top) and after (bottom) vacuum drying process. Reproduced (Adapted) under the terms of the CC BY 3.0 license.^[^ ^38^ ^]^ Copyright 2018, the Royal Society of Chemistry; c) Morphology of the FAPbI_3_ perovskite films on ZnO/PEIE substrates. The scale bar is 1 µm; d) Illustration of the domain facilitated light extraction in the structure of PeLED. Reproduced with permission.^[^ ^95^ ^]^ Copyright 2018, Springer Nature; e) Sample structure and photos of the Cs_x_FA_1‐x_PbI_3_ films; f‐g) UV‐vis absorption and PL spectra of Cs_x_FA_1‐x_PbI_3_ films deposited on different metal oxide substrates. Reproduced (Adapted) under the terms of the CC BY 4.0. license.^[^ ^37^ ^]^ Copyright 2019, Springer Nature.

However, extra amounts of A‐site cation salts in the perovskite precursors changed the film crystallization processes and promoted the formation of low‐dimensional perovskite or yellow phase (δ phase), which limited their charge injection and transporting properties.^[^ ^37^, ^97^ ^]^ Using basic metal oxides‐ZnO are proven able to efficiently deprotonate the extra A‐site cations and alter the perovskite film growing process in a distinctive way. Previously, we reported that basic surface feature of ZnO substrates promoted the formation of MAPbI_3_ films prepared with over‐stoichiometric precursors. The basic ZnO surface induced the deprotonation of MA^+^ at the interface and most of the generated MA gas evaporated from the films at thermal annealing processes, resulting in greatly enlarged 3D perovskite domains and much enhanced crystallinity (**Fgure** **3**a,b). While the same film compositions prepared on SnO_2_ and TiO_x_ substrates having a lower IEP preserved the original intermediate states.^[^ ^38^ ^]^ Wang and co‐workers further demonstrated that FAPbI_3_ films prepared on ZnO/PEIE (polyethylenimine ethoxylated) substrates grew into isolated domain structure, with sizes ranging from 100–500 nm. These domains distributed homogeneously on the substrates and was proved very efficient in improving light extraction efficiency (Figure 3c,d).^[^ ^95^ ^]^

It is also important to note that the basic ZnO substrates were capable to induce deprotonation of long‐chain organic cations, like BA^+^ and PEA^+^, which were widely utilized for forming 2D or Q‐2D perovskite for optoelectronic devices.^[^ ^99^ ^]^ As mentioned above, Bai et.al. noted that the PEA_2_Cs_n–1_Pb_n_Br_3n+1_ (PEABr as long chain organic spacers) film deposited on ZnO substrate showed much less 2D features and decreased PL emission (Figure 2e,f). This deprotonation of organic spacer, on the one hand, influence perovskite film growing processes by promoting the crystallization of 3D perovskite in the films. On the other hand, the deprotonation of PEA^+^ was thermally accelerated and led to unstable perovskite films.^[^ ^79^ ^]^ Both factors dramatically changed the compositions, crystallinity and emission properties of perovskite films, suggesting that interface‐induced deprotonation process needs to be carefully considered when preparing films on reactive metal oxides.

### 3.4 A‐Site Engineering of Perovskites

However, MAPbI_3_ films deposited on ZnO NP films demonstrated fast degradation even at room temperature, because of the relatively strong acidity of MA^+^.^[^ ^34^ ^]^ This faster deprotonation process resulted in poor device performance of PeLEDs.^[^ ^100^, ^101^ ^]^ FA^+^ and GUA^+^ all show higher pKa values (Table 1) among the widely used A‐site cations and perovskite films based on them demonstrate improved chemical stability.^[^ ^102^, ^103^, ^104^, ^105^ ^]^ Therefore, mixed cation perovskites precursor with excess A‐site cation halides (FAI and CsI) was deposited on ZnO NP/PEIE substrates. Here, the PEIE layer was very thin and was not continues, so some area of the ZnO films contacts directly with the perovskite films. Similar as the case of MAPbI_3_ mentioned above, 3D Cs_x_FA_1‐x_PbI_3_ perovskite films only formed on ZnO/PEIE substrates after thermal annealing processes, as proven by the dark brown film color, strong absorption shoulder and narrow PL emission spectrum (Figure 3e–g). While films on other substrates‐TiO_x_/PEIE and SnO_2_/PEIE remained intermediate states, showing strong absorption of intermediate phases (Figure 3f).^[^ ^37^ ^]^ The interface‐induced deprotonation process converted the extra FA^+^ into organic molecules, like FA, in the perovskite emissive films, which cleared the pathway for continuous crystallization and growth of the 3D perovskites. Compared to depositing stoichiometric precursor on other substrates, this interface‐induced deprotonation process greatly slowed down the perovskite film growing process and decreased defect density in the films. In addition, the extra halides in the films created a halide‐rich condition for the growth of perovskite crystals, which was believed to be beneficial for forming highly emissive perovskites.^[^ ^106^, ^107^ ^]^ Especially, at a higher ratio of 3:1 between CsI & FAI and PbI_2_, the perovskite films demonstrated the highest photoluminescence quantum efficiency (PLQE) close to 70% at a low light excitation of ≈0.1 mW cm^2^, suggesting an extremely low defect density in the films.^[^ ^37^ ^]^ This impressively high PLQE of the perovskite films led to a peak external quantum efficiency (EQE) of PeLEDs close to 20%, which was among the highest reported at that time.^[^ ^37^ ^]^

**Figure 4:**
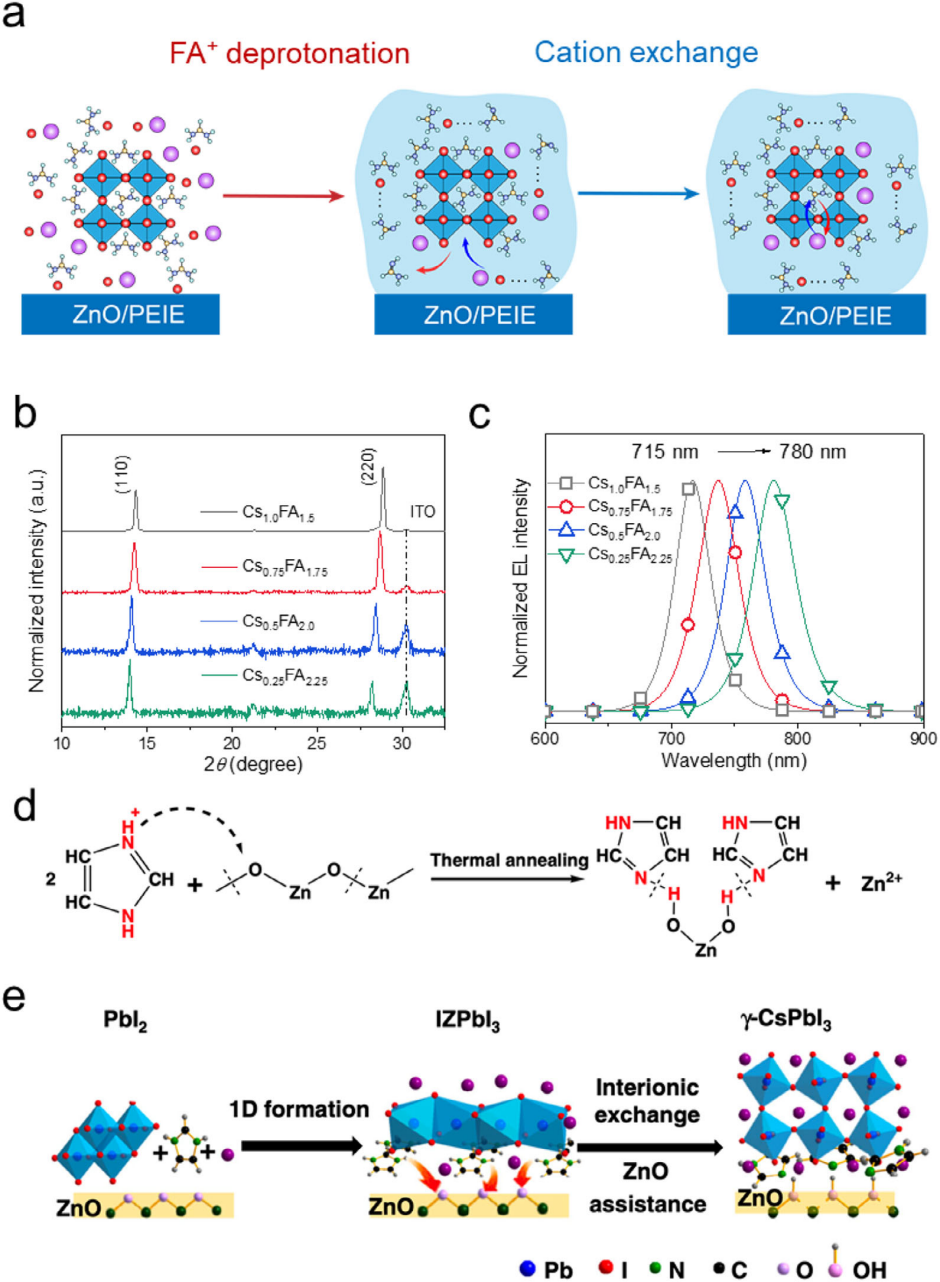
a) Illustration of the Cs‐FA cation exchange and crystal growing process during the thermal annealing process; b,c) X‐ray diffraction (XRD) patterns of the perovskite films and normalized electroluminescence spectra of PeLEDs prepared with different amounts of CsI in the perovskite emitters. Reproduced (Adapted) under the terms of the CC BY 4.0. license.^[^ ^51^ ^]^ Copyright 2022, Elsevier Inc; d) Chemical mechanism of the deprotonation of IZ^+^ by ZnO substrate; e) Film growing process of the γ‐CsPbI_3_ on ZnO substrate. Reproduced (Adapted) under the terms of the CC BY 4.0. license.^[^ ^99^ ^]^ Copyright 2020, Springer Nature.

The interface‐induced deprotonation is further proved to promote the crystallization process of mixed cation perovskite (Cs_x_FA_1‐_xPbI_3_) films and suppress the phase separation between Cs^+^ and FA^+^ based components. Conventionally, the amount of Cs^+^ should be delicately controlled to avoid phase separation in Cs_x_FA_1‐_xPbI_3_ films due to the distinct crystallization processes of Cs and FA‐based perovskites.^[^ ^108^, ^109^ ^]^ By depositing the mix cation perovskite on ZnO/PEIE substrates, the ZnO‐induced deprotonation process created an FA‐CsI hydrogel surrounding the formed perovskite domains during thermal annealing process.^[^ ^51^, ^110^, ^111^ ^]^ The continuous deprotonation of FA^+^ caused A‐site vacancies at the surface area of the perovskite crystals, while the FA‐CsI hydrogel provided the channel for transferring Cs^+^ to these vacancy sites, where the cations exchange process happened. This unique interface‐induced cation exchange process contributed to the uniformly distribution of Cs‐FA cations in the formed perovskite crystals (**Figure** **4**a,b).^[^ ^51^ ^]^ Previously, solvents or ligands assisted Cs‐FA cation exchange process was demonstrated in perovskite QDs. However, achieving uniform Cs‐FA cations distribution in the formed perovskite crystals is still very challenging.^[^ ^112^ ^]^ The interface‐assisted cation exchange process happened during the film growing process and the thermal annealing process ensured uniform cation distributions. Furthermore, this process ensured arbitrary tuning of the Cs amounts in the formed mixed cation perovskites and the fine‐tuning emission range in the 700–800 nm region (Figure 4c).^[^ ^51^ ^]^

A similar ion exchange process induced by ZnO interface was also reported in another work. To deposit CsPbI_3_ films, Yi et.al. introduced imidazolium iodide (IZI) to form an intermediate phase of IZPbI_3_ at the beginning of the thermal annealing process. During the annealing process, ZnO substrates induced the deprotonation of imidazolium cations (IZ^+^), which promoted the efficient cation exchange between Cs^+^ and IZ^+^ (Figure 4d). Therefore, highly crystalized and emissive γ‐CsPbI_3_ films could form at a relatively low annealing temperature (Figure 4e).^[^ ^99^ ^]^ Importantly, it was further verified that the organic cations, including BA^+^, hexylammonium (HA^+^), PEA^+^, and naphthylethylammonium (NMA^+^) all promoted the phase formation of γ‐CsPbI_3_ on ZnO substrates. These results indicated that the interface‐induced cation exchange process could be a general approach for preparing high quality γ‐CsPbI_3_ films for PeLEDs.

### 3.5 ZnO Substrates Engineering

There were many trials to control the interfacial reactions by doping ZnO with other metals to tune the IEP point, modifying the ZnO surface to decrease the reactivity and even isolating the direct contact between perovskite films and reactive metal oxides.

#### 3.5.1 Tuning the Isoelectric Point of the Substrates

**Figure 5:**
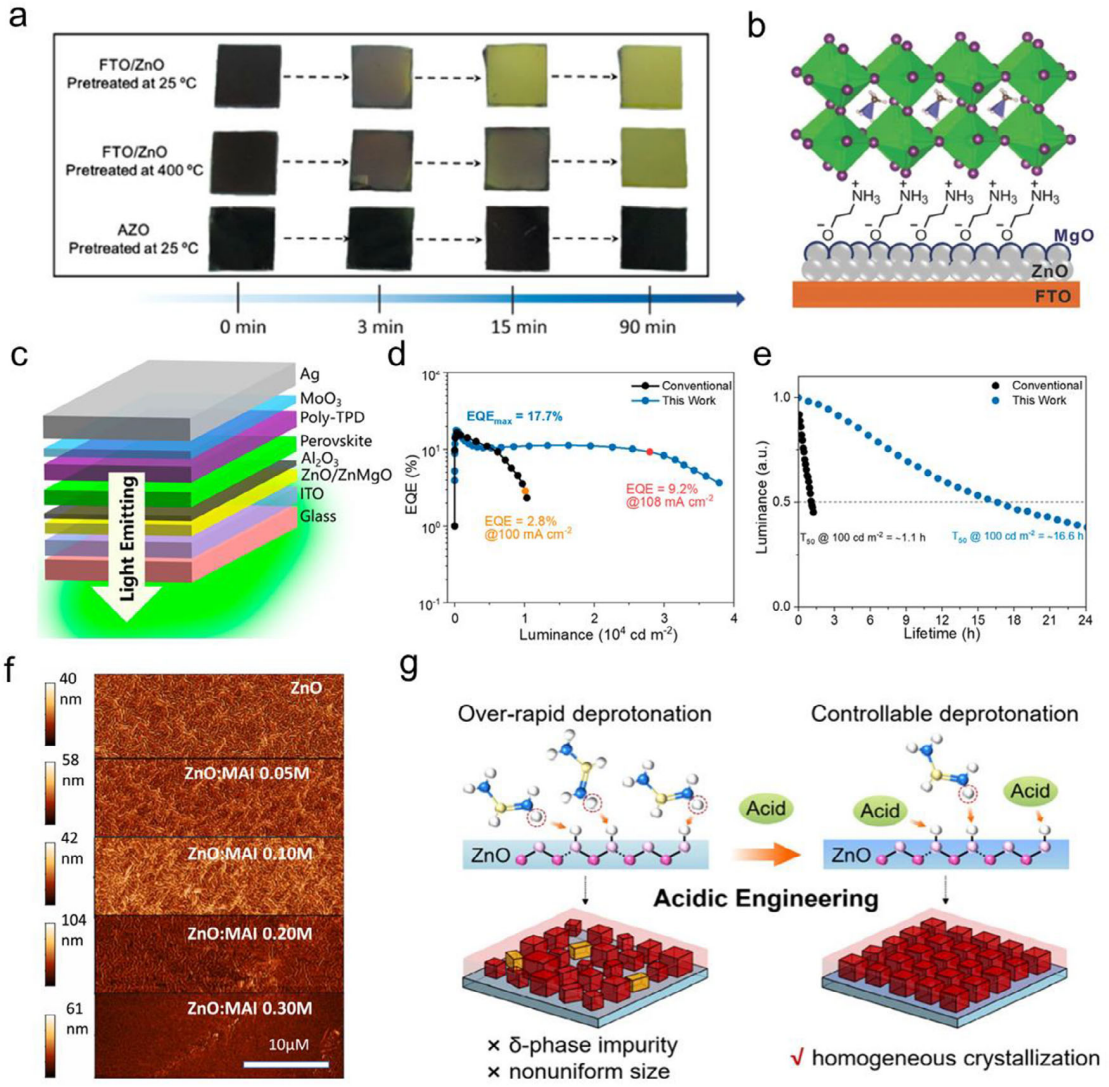
a) Influence of Al doping of ZnO films on the chemical stability of perovskite films. Reprinted with permission.^[^ ^113^ ^]^ Copyright 2016, American Chemical Society; b) Illustration of modifying ZnO surface with MgO and protonated ethanolamine (EA) molecules. Reproduced with permission.^[^ ^126^ ^]^ Copyright 2018, John Wiley and Sons; c) PeLEDs geometry on Al_2_O_3_ modified ZnO or ZnMgO substrates; d) EQE‐ current density curves and e) device operational lifetime of the PeLEDs. Reprinted with permission.^[^ ^79^ ^]^ Copyright 2022, American Chemical Society; f) Atomic force microscopy (AFM) images of ZnO substrates after MAI surface treatment. Reprinted with permission.^[^ ^82^ ^]^ Copyright 2021, Elsevier Ltd; g) Mechanism of acidic treatment of ZnO/PEIE surface and improved CsPbI_3_ crystal growing. Reprinted with permission.^[^ ^128^ ^]^ Copyright 2025, American Chemical Society.

A higher IEP of the interface material indicates a more basic surface and a faster deprotonation rate of the deposited organic‐ammonium salt. Therefore, tuning the IEP of the substrate materials is efficient in controlling the reaction rate. Doping basic metal oxides with other metal elements is an efficient approach to adjust the IEP. Zhao et.al. used aluminum‐doped zinc oxide (AZO (2 wt.% of Al_2_O_3_)) NP to replace ZnO NP for perovskite film deposition. They observed the IEP of the AZO films declined from 10.6 to 8.6, which greatly slowed down the deprotonation rate of MA^+^, consecutively improved the chemical stability of the MAPbI_3_ films (**Figure** **5**a).^[^ ^113^, ^114^ ^]^ Another work reported that by doping ZnO NPs with 10% manganese (Mn), the IEP of the Mn:ZnO NPs films dropped from 9.5 to 8.2. Both perovskite compositions of MAPbI_3_ and Cs_x_FA_1‐x_PbI_3_ demonstrated much enhanced chemical stability and extended operational lifetime of PSCs under N_2_ atmosphere.^[^ ^115^ ^]^ Zirconium (Zr) dopped ZnO could decreased the IEP from 9.3 (ZnO) to 5.32 (Zn_0.1_Zr_0.9_O), suggesting a wider tuning window is possible via simple chemical doping process.^[^ ^116^ ^]^ For p‐type metal oxides, vanadium (V) doping could reduce the IEP of NiO_x_ films to mediate the Lewis acid‐base reactions happened at the perovskite and CTMs interface.^[^ ^117^ ^]^

The doping approaches, however, might dramatically change the work function and charge carrier mobilities of these CTM films.^[^ ^118^, ^119^ ^]^ Suitable dopants and concentrations need to be delicately controlled to avoid losing the unique merits of these metal oxides.

#### 3.5.2 Interface Modifications

Modifying the top surface of the metal oxides was efficient to decrease the influence of the substrate's reactivity. There were several works conducting surface modification of metal oxide by PEIE,^[^ ^5^, ^6^, ^37^, ^38^, ^95^, ^120^ ^]^ polyvinylpyrrolidone,^[^ ^121^, ^122^, ^123^, ^124^ ^]^ polyvinyl alcohol^[^ ^125^ ^]^ etc. that contributed to much improved optoelectronic device performance. Cao and co‐workers demonstrated that a thin layer of MgO films (the surface of MgO was further treated with ethanolamine) on ZnO substrates obviously decreased the surface reactivity. The improved the operational stability of PSCs (Figure 5b) was similar to that of PSCs based on TiO_2_ substrates.^[^ ^126^ ^]^ Furthermore, Zou et.al deposited a thin and compact Al_2_O_3_ film by ALD between the perovskite emitters and the ZnO substrates to avoid their direct contact (Figure 5c). This strategy contributed to much decreased rolling‐off behaviour in the EQE curves and improved operational stability of the green PeLEDs, both of which indicating much enhanced chemical stability of the perovskite films on Al_2_O_3_/ZnO substrates (Figure 5d,e).^[^ ^79^ ^]^

It was interesting to note that treating basic metal oxide surface with acidic materials could mediate the surface reactivity through MAI,^[^ ^82^ ^]^ FAI,^[^ ^105^ ^]^ 4‐fluoro‐phenethylammonium iodide (*p*‐F‐PEAI) and MACl treatment.^[^ ^41^, ^127^ ^]^ Tsarev et.al. have shown that by treating the ZnO NPs films with extra amounts of MAI, the *in‐situ* deprotonation of MA^+^ induced by ZnO substrates converted the top surface of ZnO films to ZnI_2_. This transformation could be further observed from the greatly changed surface morphology of the ZnO substrates as shown in Figure 5f. Thereafter, the thermal stability of both MAPbI_3_ and Cs_0.12_FA_0.88_PbI_3_ films on ZnO substrates dramatically enhanced.^[^ ^82^ ^]^ Zhao and co‐workers performed a more dedicated study on acid treatment of ZnO NP films (Figure 5g).^[^ ^128^ ^]^ Among all the acidic materials with various pKa, molecules with mild acid dissociation constants mitigated the deprotonation rate of FA^+^ efficiently by decreasing the reactivity of the ZnO substrates. This further led to retarded crystallization process and a more homogeneous crystallinity in the CsPbI_3_ films. By selecting 1,4‐cyclohexanedicarboxylic acid, a molecule with a pKa of 4.43, the PeLEDs achieved the highest EQE closeing to 20% with a much‐improved emitting brightness.^[^ ^128^ ^]^

## 4 Stabilizing Perovskite Films on ZnO Substrates

Though the interface‐induced deprotonation process of organic cations facilitated the crystallization of 3D perovskites, after device fabrication, the continued deprotonation process caused the collapse of the perovskite lattice and ensuing device degradation.^[^ ^24^, ^37^ ^]^ Therefore, PeLEDs based on ZnO substrates commonly showed an operational lifetime within a few hours (without introducing special additives).^[^ ^6^, ^37^, ^99^ ^]^ The interface‐induced deprotonation process of organic cations was believed to be one of the main factors hindering the performance of PeLEDs.^[^ ^24^ ^]^ To further enhance device operational lifetime, strategies for inhibiting the interfacial reactions after device fabrication are urgently required.

### 4.1 Interfacial Induced Amidation Process

**Figure 6:**
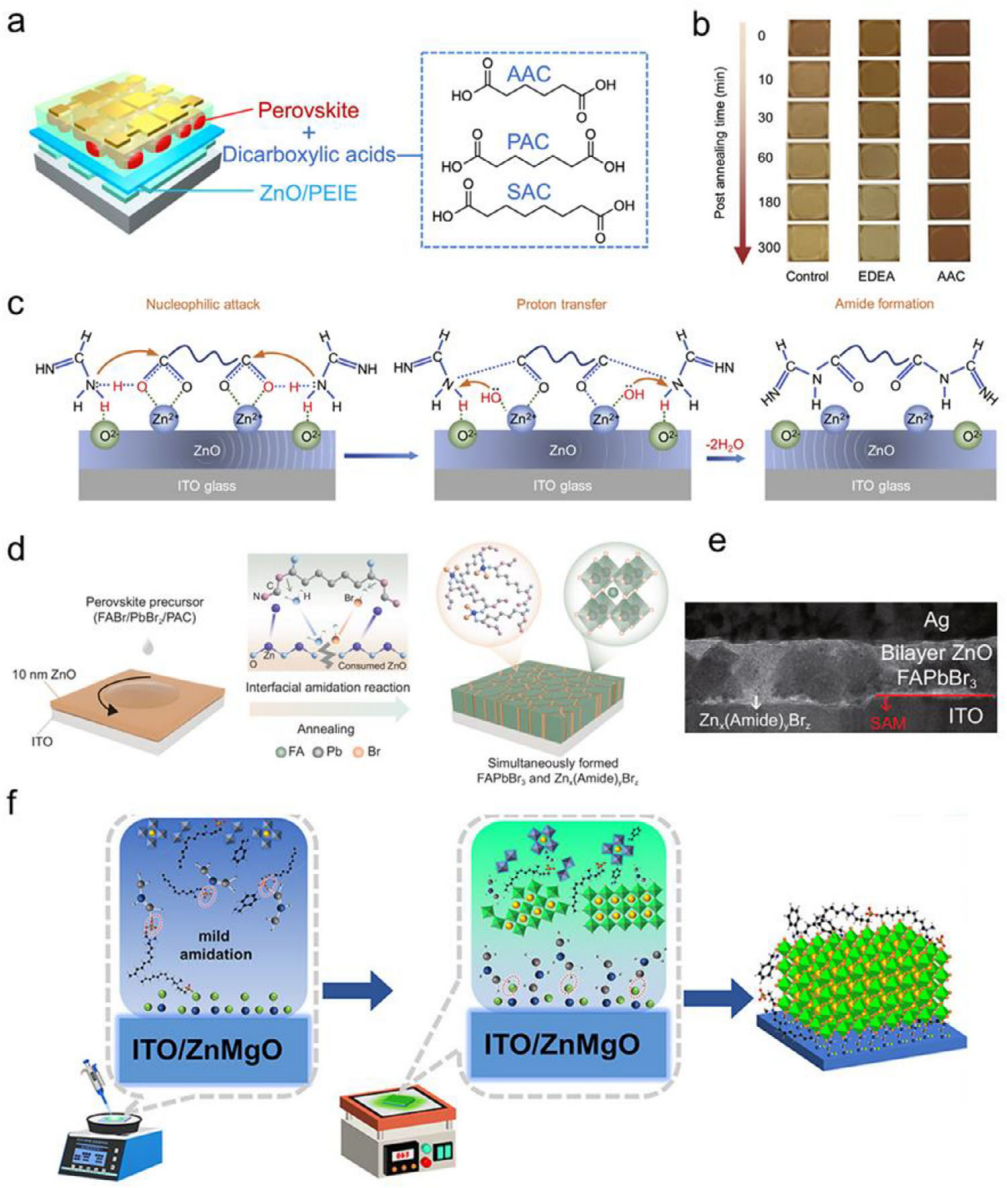
a) Device structure of PeLEDs and molecular structure of dicarboxylic acids used in the perovskite precursors; b) Images of the perovskite films (control films and films with EDEA and AAC as additives) during thermal stress; c) Schematic illustration of the interface induced amidation reaction during the perovskite film growing process. Reproduced (Adapted) under the terms of the CC BY 4.0. license.^[^ ^24^ ^]^ Copyright 2021, Elsevier Inc.; d) Schematics illustration of the amidation process and the formation of the matrix perovskite emitting layer; e) Cross‐sectional scanning transmission electron microscope image of the all‐inorganic device structure. Reproduced (Adapted) under the terms of the CC BY 4.0. license.^[^ ^130^ ^]^ Copyright 2025, Springer Nature; f) Mechanism of the enhanced crystallinity of FAPbBr_3_ films on ZnMgO substrates with SFB as the processing additive. Reprinted with permission.^[^ ^132^ ^]^ Copyright 2025, American Chemical Society.

Improving the chemical stability of perovskite films is a prerequisite for highly stable devices. In one of our works, we introduced a dicarboxylic acid – adipic acid (AAC), into the precursors of FAPbI_3_ for preparing highly stable PeLEDs.^[^ ^24^ ^]^ This additive greatly improved the thermal stability of FAPbI_3_ film on ZnO substrates comparing to that of the control films and films prepared with 2,2′‐(ethylenedioxy) diethylamine (EDEA) as an additive (**Figure** **6**a). After 300 min thermal annealing process, no color change was observed for the AAC incorporated films (Figure 6b). During the thermal annealing process, ZnO catalyzed an in‐situ amidation process between dicarboxylic acids additives and the excess FA^+^ in the perovskite films (Figure 6c). This *in‐situ* interfacial reaction, on the one hand, converted the reactive organic cations (FA^+^) into inert amides and fixed the FA components in the perovskite films. Furthermore, the amides isolated the direct contact between ZnO substrate and FA^+^, which further hindered FA^+^ deprotonation process after the device fabrication processes, therefore stablized the interfaces. Both factors greatly prolonged the operational lifetime of PeLEDs to 179 and 682 h, when AAC and pimelic acid (PAC) were utilized as the processing additives, respectively.^[^ ^129^ ^]^ This interface induced amidation strategy was further applied in green PeLEDs with FAPbBr_3_ as emitter. By depositing FAPbBr_3_ precursor with PAC as a processing additive on 10 nm ZnO film, the amidation process between PAC and FA^+^ was successfully triggered by ZnO substrate. It was believed that the byproduct of this amidation interacted with Zn^+^ and etched the ZnO films, leading to the formation of a supramolecular metal complex (Zn_x_(Amide)_y_Br_z_) matrix around the FAPbBr_3_ domains (Figure 6d,e). This special matrix structure remarkably enhanced the thermal stability and PLQE of the FAPbBr_3_ films. The ensuing PeLEDs demonstrated a maximum EQE of 18.2%, a highest brightness of 312 000 cd m^2^ and the device operational lifetime (T_50_) was improved to 350 h (at an initial brightness of 1000 cd m^2^).^[^ ^130^ ^]^

The interface‐induced amidation was further verified happening between the ligands on the substrate surface and the organic cations in perovskite emitters. By depositing FAPbBr_3_ film on sol‐gel prepared ZnO substrates, Du et.al. revealed that the surface carboxylate ligands on ZnO surface reacted with FA^+^ in the perovskite films, forming an amino‐rich interface on top of ZnO substrate and enhanced the crystallinity of FAPbBr_3_.^[^ ^131^ ^]^ Further incorporating more magnesium acetate increased the acetate ligands density on the formed ZnMgO films surface. It created more sites at the ZnMgO/FAPbBr_3_ interface for amidation reaction and dramatically enhanced films crystallinity and emission property, leading to a highest PLQE to more than 60%.^[^ ^131^ ^]^ In addition to the density of amdiation sites, the reaction rate could be controlled by adding additives in the perovskite precursors. Because of the strong interaction between caprylyl sulfobetaine (SFB) and FABr, SFB decelerated the amidation process between the carboxylate groups on the substrates and FABr, leading to much enhanced crystallinity of the FAPbBr_3_ films (Figure 6f). The obtained PeLEDs showed the highest EQE of over 20% and a maximum luminance of 120, 000 cd/m^2^, which was the most efficient bromine‐based PeLEDs with perovskite films directly deposited on ZnO films.^[^ ^132^ ^]^

### 4.2 Controllable Interfacial Induced Deprotonation Process

**Figure 7:**
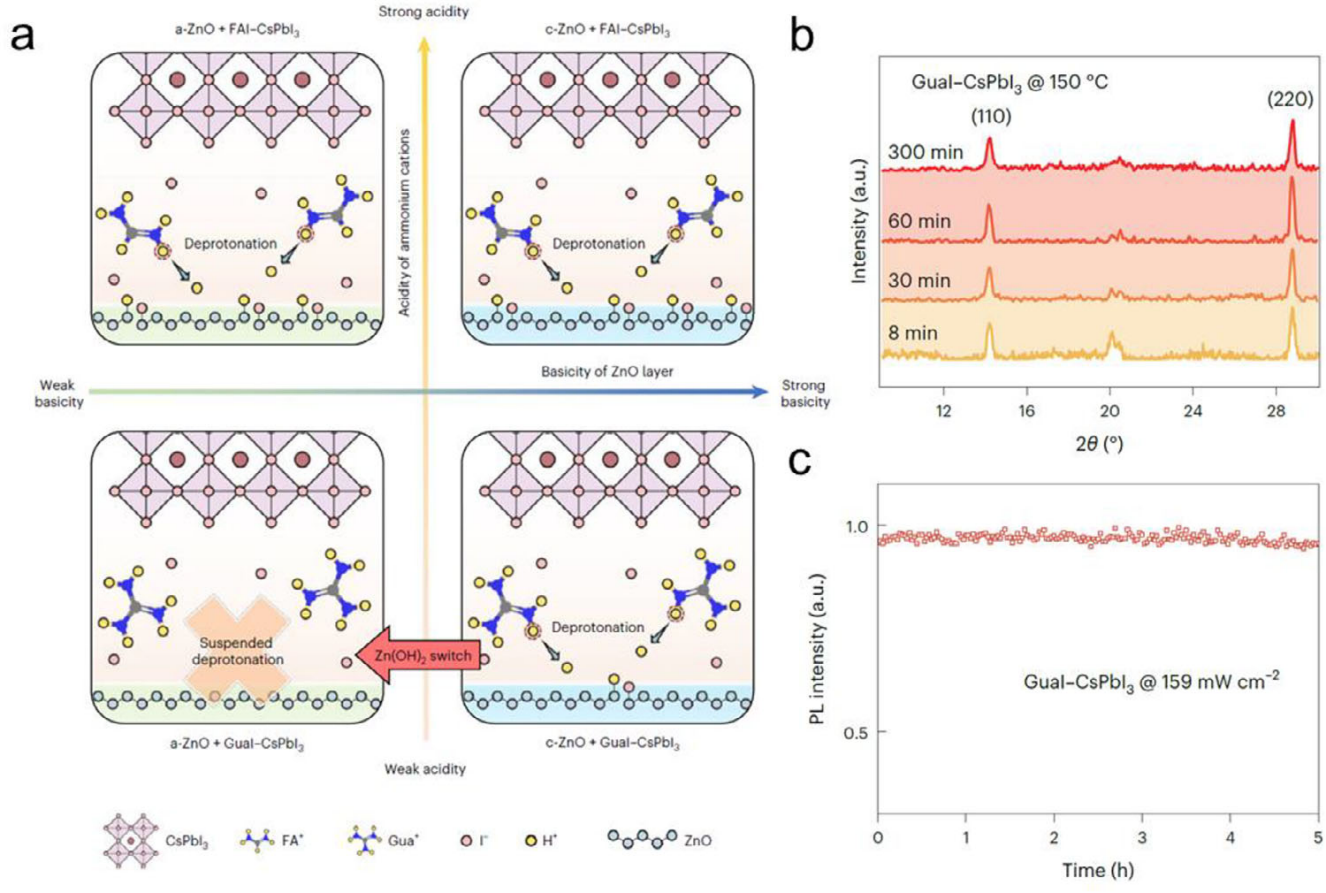
a) Mechanism of the switchable interfacial deprotonation reactions; b) XRD patterns of GUAI‐CsPbI_3_ deposited on Zn(OH)_2_ substrates annealed at 150 °C for various durations, c) PL intensity stability of the GUAI‐CsPbI_3_ film under a continuous 515 nm laser (159 mW cm^−2^). Reproduced with permission.^[^ ^36^ ^]^ Copyright 2024, Springer Nature.

Terminating interfacial reactions after perovskite film deposition is necessary to avoid the detrimental effects on the operational stability of perovskite optoelectronic devices. As discussed above, the rate of interface‐induced deprotonation process depended on the *pKa* value of the organic cations and IEP values of the substrates. Zeng et.al. developed a switchable interfacial deprotonation to achieve highly stable PeLEDs.^[^ ^36^ ^]^ In this strategy, GUA^+^ possessing a higher pKa value compared to FA^+^, was used as organic cations in Cs based perovskite emitters (Table 1).^[^ ^35^ ^]^ The commonly prepared ZnO NP were not basic enough to deprotonate GUA^+^ during the perovskite film deposition process. To deprotonate GUA^+^, Zn(OH)_2_ film with a higher basicity was prepared from an aqueous solution of ammine‐hydroxo zinc complex (Zn(NH_3_)_n_(OH)_2_). At a lower annealing temperature of 60 °C, the Zn(NH_3_)_n_(OH)_2_ film lost ammonia and generated thin Zn(OH)_2_ film, which became ZnO films after a higher annealing temperature via dehydration reaction (**Figure** **7**a).^[^ ^133^ ^]^ The authors creatively use this Zn(OH)_2_ film to “switch on” the deprotonation GUA^+^, due to the higher basicity of Zn(OH)_2_ film. After the deprotonation reaction, Zn(OH)_2_ film was converted to ZnO film, which was no longer able to deprotonate the GUA^+^, and thus “switch off” the deprotonation reaction (Figure 7a). Accordingly, the formed CsPbI_3_ perovskite film exhibited greatly improved thermal and PL stability during thermal annealing process and at a high excitation intensity (159 mW cm^−2^), respectively (Figure 7b,c). It is important to note that the PeLEDs exhibited a greatly extended operational lifetime of around 33 h at a constant driving current density of 100 mA cm^2^. This work provided a clearer insight into controlling the interface‐induced deprotonation for high‐performance PeLEDs.^[^ ^36^ ^]^

### 4.3 Interface Modification Layers Induced Reactions

**Figure 8:**
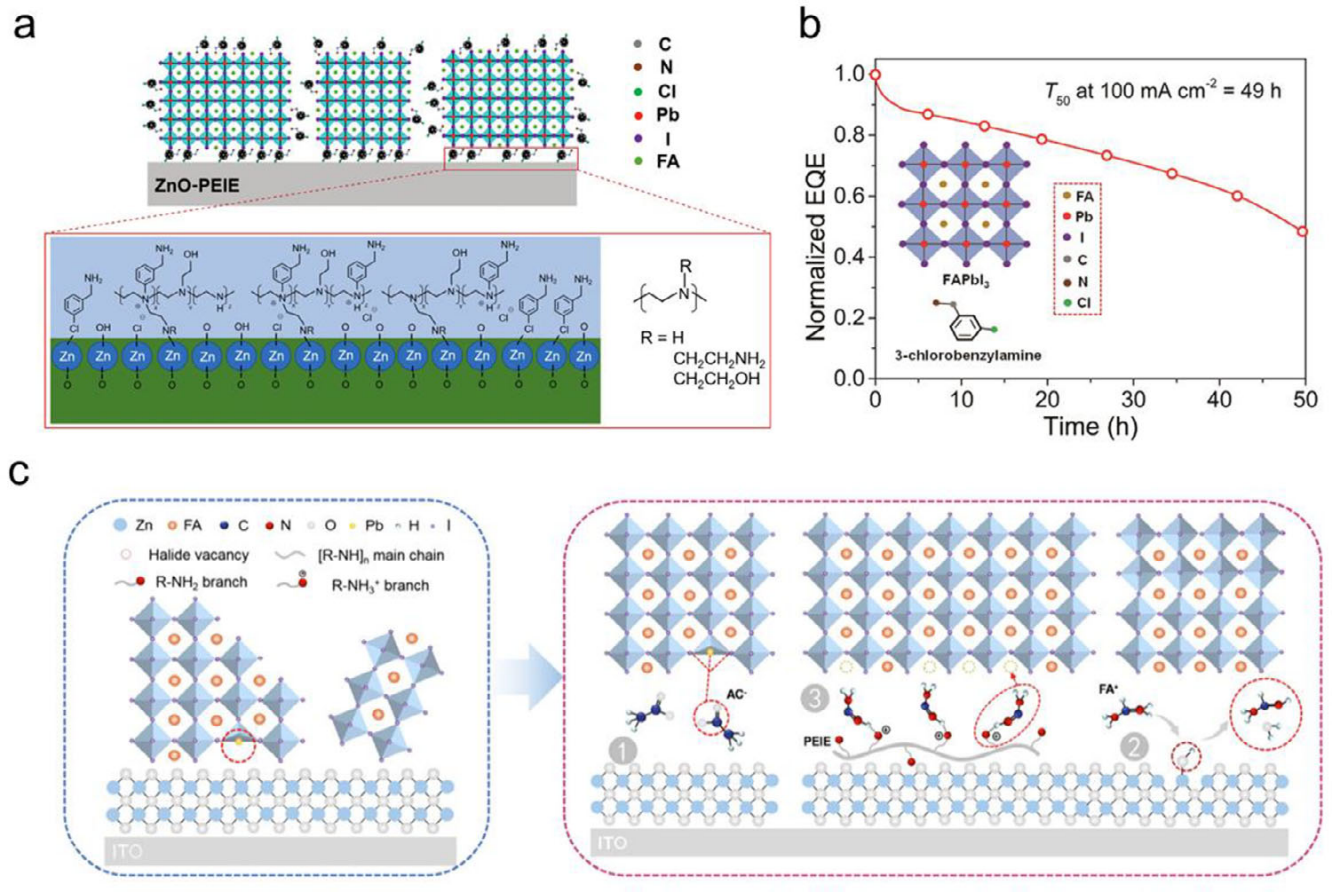
a) Schematic illustration of 3Cl‐BA‐induced perovskites crystallization process; b) Operational lifetime of PeLEDs using 3Cl‐BA as processing additive. Reprinted with permission.^[^ ^134^ ^]^ Copyright 2021, American Chemical Society; c) Mechanism of the multifunctional FAAc surface treatment: Process 1‐ Ac^‐^ groups passivating the defects of perovskites; Process 2‐mediating ‐OH defects in ZnO films via interfacial induced deprotonation of FA^+^; Process 3‐protonation transfer reaction between PEIE and FA^+^. Reprinted with permission.^[^ ^135^ ^]^ Copyright 2024, American Chemical Society.

Chemical reactions between perovskite precursors or processing additives and the modification layers of metal oxides were another vital factor influencing the perovskite films growing process. Y. Cao et.al revealed that when depositing FAPbI_3_ films on ZnO/PEIE substrates with 5‐amino valeric acid (5AVA) as the processing additive, a dehydration reaction happened between 5AVA and PEIE modification layer. This interfacial reaction created an organic insulating layer between the formed perovskite domains and ZnO, contributing to a much‐decreased leakage current in the PeLEDs.^[^ ^95^ ^]^ The PeLEDs demonstrated impressive EQE of over 20% and an operational lifetime of 20 h (driving current density 100 mA cm^2^). The same team also introduced a processing additive 3‐chlorobenzylamine (3Cl‐BA) in perovskite precursor. Importantly, 3Cl‐BA formed coordinating bonds with ZnO and PEIE layer via Cl side during film deposition, leaving the other ‐NH_2_ side as nucleation sites for perovskite crystals. This unique arrangement of 3Cl‐BA on the ZnO/PEIE substates induced more orientated growth of FAPbI_3_ from the bottom interface, leading to improved crystallinity and reduced defects density of FAPbI_3_ emitters (**Figure** **8**a). This strategy efficiently suppressed the ion migration in the perovskite emitters and mediated the efficiency roll‐off behavior of the devices, contributing to extended operational lifetime of around 49 h (driving current density 100 mA cm^2^) (Figure 8b).^[^ ^134^ ^]^ In addition, Chen and co‐workers placed a thin layer of formamidine acetate (FAAc) between FAPbI_3_ films and ZnO/PEIE substrates.^[^ ^135^ ^]^ They observed an *in‐situ* proton transfer reaction from FA^+^ to amino group in PEIE, which provided nucleation sites for the vertical growth of the FAPbI_3_ crystals. Additionally, this interface induced deprotonation removed the hydroxyl defects in ZnO films and improved radiative recombination in the perovskite films (Figure 8c). Due to the multifunctional role of FAAc, the FAPbI_3_‐based PeLEDs demonstrated the highest EQE of 23.84% and a half lifetime of 24 h. The same *in‐situ* proton transfer reaction happening between FA^+^ and PEIE was also verified efficient in reducing deep‐level traps at the surface of the perovskite films and contributed enhanced operational stability of p‐i‐n PSCs.^[^ ^136^ ^]^

## 5 Outlook

**Figure 9:**
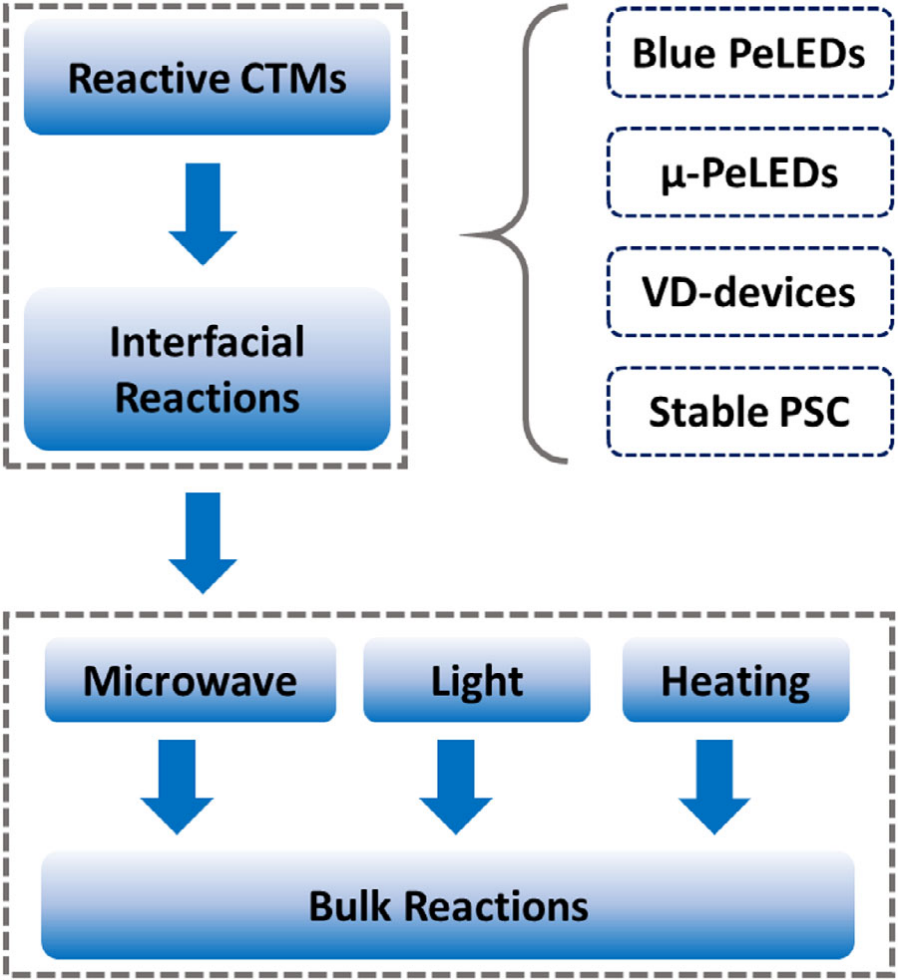
Schematic illustration of the proposed strategies.

Interfacial reactions have been identified as unique factors influencing the optoelectronic properties of perovskite films, and they are now widely utilized in PeLEDs emitting in the NIR, red, and green regions.^[^ ^137^ ^]^ We believe they hold great promise for further advancing the development of high performance blue PeLEDs, micro‐PeLEDs, vacuum deposited perovskite optoelectronic devices and even highly stable PSCs (**Figure** **9**).

### 5.1 Blue PeLEDs

It is very attractive to utilize metal oxides like, ZnO or ZnMgO as CTMs in blue PeLEDs since devices based on them commonly demonstrate low turn‐on voltages (the lowest driving voltage that needed to turn on devices to reach a certain brightness) and high brightness, as compared to those based on organic CTMs. A lower driving voltage minimizes the electric field across the device, which eases the ion migration and could be vital for further enhancing the operational stability of PeLEDs.^[^ ^130^, ^138^ ^]^ Additionally, several works revealed that treating ZnO surface with chloride (Cl^‐^) ions decreased the reactivity and defect density at ZnO surface.^[^ ^127^, ^139^ ^]^ However, high‐performance bule PeLEDs with ZnO as CTM have rarely been reported. This suggested that for blue perovskite emitters, the interfaces might play even more important roles as compared to the case of NIR PeLEDs, and new designs of interfacial reactions might be required. For example, introducing new processing additives and/or developing novel methods to modify the reactive metal oxide surface would significantly enhance the emission properties of blue perovskite emitters and contribute to the realization of highly efficient and stable blue PeLEDs ^[^ ^131^ ^]^

### 5.2 Micro‐PeLEDs

Interfacial reactions showed great impacts on the growing process of perovskite emitters, resulting in the isolated and well passivated perovskite domains.^[^ ^95^ ^]^ These perovskite domains with submicrometre‐scale, on the one hand, enhanced light extraction efficiency in the PeLEDs. One the other hand, they would be very promising for achieving high performance micro‐PeLEDs (µ‐PeLEDs) that requiring emitting pixel sizes below 100 µm (even below 1 µm) for applications in augmented or virtual reality. The surface area of the isolated perovskite domains (size from 50–1000 nm) was well passivated and would be able to dramatically decrease the edge‐induced non‐radiative recombination when miniaturizing LEDs pixels.^[^ ^140^, ^141^, ^142^ ^]^

### 5.3 Vacuum Deposited Perovskite Optoelectronic Devices

In addition to solution processes, vacuum deposition techniques have shown great promise for large‐scale production of perovskite‐based devices.^[^ ^143^, ^144^ ^]^ Compared to solution‐processed devices, substrate interface plays an even more important role in the crystal nucleation and films growing processes of vacuum deposited (VD) films and devices.^[^ ^145^, ^146^, ^147^, ^148^ ^]^ Yan et.al. explored depositing thin templating perovskite layers on various substrates for the growing of co‐evaporated perovskite films. This strategy greatly minimized the influence of interfacial materials, highlighting the importance of interface modification for vacuum deposited perovskite films.^[^ ^149^ ^]^ Further exploring vacuum deposited interfacial materials and controlling the interface reactions could be important approaches to adjusting the optoelectronic properties of perovskite films on different substrates.

### 5.4 Stable PSCs

Terminating the interfacial reactions after the device preparation can be critical for achieving more stable PeLEDs.^[^ ^79^ ^]^ The work of switchable interfacial reaction was a very good example showing the interface‐induced reactions could be well managed.^[^ ^36^ ^]^ Following similar strategy, highly stable PSCs might be achieved as a stable interface is a must. It is important to note that the interface‐induced deprotonation process greatly minimize the defect density both in perovskites and interfacial materials, resulting in improved emission efficiency of PeLEDs. Utilization of a similar strategy in PSC might remarkably decrease the defect density related to open circuit voltage loss (*V_oc_* loss), contributing to further improved power conversion efficiency in PSCs. However, this may require a deeper understanding of the mechanism of interface reactions to avoid the negative effects.

### 5.5 Moving from the Interface to the Bulk

Currently, the chemical reactions discussed in this review are primarily induced by the CTMs at the interfacial area, and these reactions may damage the charge transporting properties of the CTMs or alter the energy level alignment across the device. Therefore, exploring more interfacial modification materials to protect the metal oxides' surface could be vital to further improve the quality of perovskite films and the optoelectronic device performance. For example, introducing conjugated polyelectrolytes could be efficient to modify the reactive surface of metal oxide and maintain the outstanding electronic properties.^[^ ^150^ ^]^

Further moving the interfacial chemical reactions to the bulk of perovskite films, or even inducing the reactions in the preparation stage of the perovskite precursors before film deposition processes, could be a more efficient approach for better reaction control. Accordingly, the reaction could be triggered dramatically different from the factors we discussed above. Other factors, including light, heating or microwave could be of great interests to induce the chemical reactions for perovskite film growing.^[^ ^151^, ^152^, ^153^, ^154^, ^155^, ^156^ ^]^ An obvious advantage of these inducing methods is that the reactions can be easily terminated after film deposition, which is ideal to avoid their negative impacts on the chemical stability of perovskite films and CTMs. Furthermore, the chemical reactions summarized in this review are mainly based on proton transfer or acid ‐base reaction, amidation and dehydration, etc., more reactions including polymerization, condensation or hydrolysis could also be used to control the perovskite film growing process and/or achieve efficient defect passivation.

## 6 Conclusion

Interfacial reactions frequently occur in perovskite‐based optoelectronic devices during device fabrication and operation. Different from the view that interfacial reactions deteriorate the device performance, they show very positive roles in controlling perovskite compositions, defect formation and passivation during perovskite films growing. And careful control of the interfacial reactions would be a key factor for obtaining high performance perovskite based optoelectronic device. However, further strategies on limiting the negative impacts of the interfacial reactions on device performance, especially on operational stability are urgently needed. Moving the reaction location to the bulk of perovskite films, or even inducing the reactions prior to film deposition process could be of great interests to explore. Additionally, the interfacial reactions may also inspire new strategies for preparing high‐performance optoelectronic devices based on inorganic QDs, organic materials and other functional materials.

## Conflict of Interest

The authors declare the following competing interests: H.J.S. is a co‐founder of Oxford PV and Helio Display Materials, which are companies commercializing perovskite‐based photovoltaics and light‐emitting applications, respectively. The other authors declare no competing interests.
